# Evaluation of Hearing Results in Otosclerotic Patients after Stapedectomy 

**Published:** 2011

**Authors:** Nader Saki, Soheila Nikakhlagh, Mahmood Hekmatshoar, Narges Mofrad Booshehri

**Affiliations:** 1 *Department of otorhinolaryngology, Ahwaz Jundi ShapourUniversity of Medical Sciences, Ahwaz, Iran*; 2 *Department of otorhinolaryngology, Ahwaz Jundi ShapourUniversity of Medical Sciences, Ahwaz, Iran*; 3 *Department of otorhinolaryngology, Ahwaz Jundi ShapourUniversity of Medical Sciences, Ahwaz, Iran*; 4 *General physician, Ahwaz Jundi ShapourUniversity of Medical Sciences, Ahwaz, Iran*

**Keywords:** Complications, Hearing loss, Otosclerosis, Stapedectomy

## Abstract

**Introduction:**

Otosclerosis one of the most common causes of conductive hearing loss is more common in females and in their middle ages. It is usually a bilateral and progressive disease. Surgical operation is suggested as the exclusive management of otosclerosis. This study aims to evaluate the hearing results after stapedectomy in otosclerotic patients in Ahwaz.

**Materials and Methods:**

In this case series study, the records of otosclerotic patients who had undergone stapedectomy or stapedotomy in Imam Khomeini and Apadana Hospitals of Ahwaz, Iran during 1997-2007 were evaluated. All the operations were performed by a single surgeon (first author). Data were analyzed using SPSS and descriptive statistical tests.

**Results:**

One hundred ninety seven patients were included in this study. 66.8% were female and the age range was 20-40 years. The affected ears were reported as follows: right ear (65%), left ear (35%) and bilateral (18%). ABG was reported as less than 10db in 63.9% of patients; between 10 to 20db in 29.99% and more than 20db in 5%. Surgical complications were observed in 4.5% of patients (1.5% intraoperatively and 3% postoperatively).

**Discussion:**

Saccular dysfunction seems to be an important finding in SSNHL. Although it is more prevalent in the patients with vertigo, it can be found in the non-dizzy cases. VEMP disturbance in SSNHL shows more extensive pathological involvement.

**Conclusion:**

Just like previously conducted studies, satisfactory surgical outcomes with rare complications were observed in the appropriate population under study.

## Introduction

Otosclerosis is one of the most common causes of conductive hearing loss in people with 15-50 years of age. Otosclerosis has been derived from a Greek word meaning ear hardening.

The disease now refers to the bony ear capsule and may result in progressive and conductive hearing loss, mixed hearing loss and also absolute sensorineural hearing loss in some rare cases (1). Otosclerosis is an hereditary disease which is transmitted in an autosomal dominant form with incomplete penetrance (1). 

Bilateral otosclerosis has been observed in 60% of patients (2). Otosclerosis occurs most commonly among Caucasions with an incidence of 1% followed by Asians at 0.5%, African-American men at 7.3% and African-American women at 10.3% (1). Progressive and conductive hearing loss particularly in low frequencies (500-2000 Hz) which may sometimes occur with sensorineural hearing loss has been identified as the main clinical finding of otosclerosis (1). Tinnitus has been reported as the other common symptom of this disease (2). There is not any definitive medical treatment for the disease; however, some surgical methods such as stapedectomy or stapedotomy may be effective in the treatment of hearing loss. Stapedectomy includes removal of the stirrup, creating a small bone in the Foot plate and inserting prosthesis in between the incus and the oval window (3). The related complications of stapedectomy include: sensorineural hearing loss, dizziness, facial nerve paralysis, tinnitus, degradation of taste, eardrum perforation, perilymph otorrhea and perilymph fistula. Delayed treatment may lead to otosclerosis progression and permanent deafness; so the disease should be diagnosed in early stages and stapedectomy has to be performed as the treatment of choice for improvement of this disease (2). 

In order to specify whether the optimal surgical outcome has been obtained and whether it has had any significant effect on returning the patients' hearing or not; patients should be evaluated postoperatively regarding their hearing results. This study aims to evaluate and compare the improved hearing results after stapedectomy in otosclerotic patients referred to Imam Khomeini and Apadana Hospitals of Ahwaz with the global scale.

## Materials and Methods

In this case series study, otosclerotic patients who had undergone stapedectomy and stapedotomy in Imam Khomeini and Apadana Hospitals of Ahwaz, Iran during 1997-2007 were evaluated. All the operations were performed by a single surgeon (first author). Complete stapedectomy includes complete removal of the stirrup base; incomplete stapedectomy is removal of a third of the stirrup base whereas incomplete stapedotomy is creating a small opening (0.4 to 0.8 mm in diameter) in the stirrup base.The most common grafts used for covering the oval window or the created opening included fat, clot, gelfoam and perichondrium. A 6 mm prosthesis was applied in most patients. Those patients without a preoperative and postoperative ear bar were excluded from the study. Finally, 197 subjects including 105 females and 92 males were selected. Patients' records were evaluated and some data regarding their age, sex, involved ear and unilateral or bilateral otosclerosis were gathered. Patients' preoperative and postoperative audiometry tests were also analyzed and other data consisting of air conductive threshold, bone conductive threshold and air - bone gap in speech frequencies (500, 1000 and 2000 Hz) were collected. The obtained data were analyzed by SPSS (version 16). One sample T-test was applied to compare the means and unilateral ANOVA test was used for comparison between groups. Significant Pvalue was reported as less than 0.05 (*P*<0.05).

## Results

197 patients including 105 females (53.3%) and 92 males (46.7%) undergoing stapedectomy or stapedotomy were evaluated in this study. Subjects were 18-67 years old with the mean age of 38.4 years. 

98 patients (49.7%) were in the age range of 30-40 years (Table 1).

**Table 1:** Number of patients based on age/sex

**Age/Sex**	**Female**	**Male**	**Total **%	
<20	2	1	3	1.5
21-30	33	41	74	37.5
31-40	56	42	98	49.7
41-50	8	4	12	6.1
51-60	3	3	9	3.1
< 60	3	1	4	2.1
Total	105	92	197	100
				

Unilateral ear involvement was revealed in 161 patients (81.7%) and bilateral involvement in 36 (18.2%). 233 surgical operations were performed on 197 patients, from which 36 underwent bilateral surgery. Surgery on the right ear was reported in 152 (65.3%) and on the left ear in 81 cases (34.7%). The reported ABG was less than 20db postoperatively in 185 patients (93.9%). Deafness occurred in 2 patients (1%) after the surgery (Table 2).

**Table:** 

**Mean ABG postoperatively** **surgery done with 500-1000-2000 frequencies**	**Number of patients**	**Result**	**Percentage**
0-10 dB	126	Good	69.96
10-20 dB	59	Acceptable	29.96
20-30 dB	7	Weak	3.55
>10 dB	3	Not satisfactory	1.52
Deafness	2	Bad	1.01

*Air-Bone Gap (ABG)

Some significant intraoperative or postoperative complications were detected in 9 patients (4.5%). Perilymph gusher as an intraoperative complication was observed in one case (0.5%) and it was repaired immediately. Tympanic membrane perforation occurred in 2 cases (1%) intraoperatively, which was repaired by myringoplasty. 3 patients (1.5%) revealed severe postural vertigo after stapedectomy; therefore, conservative treatment was given for 4 weeks to totally improve the condition. One patient (0.5%) showed taste disorder and 2 patients (1%) suffered from complete deafness postoperatively. The mean AC of the studied cases' was reported as 55.5 db preoperatively which reached to 27.5 db after the operation. Also the patients showed a mean ABG of about 33.6db preoperatively and 11.9 db postoperatively.

## Discussion

Otosclerosis is one of the most common diseases involving the ear capsule. This disease occurs only in humans and is more common in middle aged women. Otosclerosis has been diagnosed as the most common cause of conductive hearing loss. It may even progress in to permanent deafness. Histopathologically, the otosclerosis process is divided in to two phases including the early phase and the late phase. Increased vascularity, hyperemia and bone resorption characterize the early phase. In the late phase, the reabsorbed bone is replaced with dense selerotic bone. Otosclerosis is treated with stapedectomy or stapedotomy which may result in returning the patient's hearing to normal. Stapedectomy is an elective surgery, affecting the patient and his or her relatives' quality of life. Surgeon's experience plays an important role in performing this delicate type of surgery on the stirrup. Although permanent and severe complications are mentioned for stapedectomy or stapedotomy, but fortunately they are not common.

Most patients (87.5%) included in this study were 20-40 years old. In the study performed on 475 Spanish patients, the most involved age was 15-45 years (62.2%) (4); whereas in a study on 65 English patients in 1996 it was reported as 40-49 years (5). Since Iran's population is mainly formed by the young generation, it may be the reason for the different age prevalence. Females (68.4%) included the most involved sex under study in Spain; while it was reported as 53.3% in ours. In an extensive study conducted on 2525 French patients during 1991-2004, 67% of the involved cases were female and 33% were male (6).

The statistical similarity showed that otosclerosis is more prevalent among females. Involvement of the right and left ear was reported in 56.3% and 34.7% of cases in the our study, respectively; whereas in a study carried out on 50 Brazilian patients in 1997-2000, the mostly involved ear had been the left ear (53%) (7). In study of India , 70% of the 30 studied patients showed bilateral and 30% showed unilateral involvement (8); whereas only 18.2% of the cases revealed bilateral involvement in our study. This significant difference may have been due to some missing data in the patients' records as well as lack of accurate and targeted follow-up or little follow-up in the population under study. Major surgical complications in our study were observed in 4.5% of patients (1.5% intraoperatively and 3% postoperatively); the same figure being 27.3% in Brazil (15.6% intraoperatively and 11.7% postoperatively) (7). 

In comparison with other studies, ABG results were as follows:

ABG < 10dB:

Present study: 63.9%, France: 92.4%, Slovenia: 52.4%, Brazil: 70.5%, Malaysia: 74.3% and Rawalpindi: 56.7%.

20< ABG >10 dB:

Present study: 29.9%, France: 3.8% and Brazil: 16%.

ABG >30 dB:

Present study: 2.5% including complete deafness (1%) and in Brazil: 7.8% including complete deafness (1.9%). 

Other studies revealed the same results relatively (4, 9-16). In a study on 34 Malaysian patients during 1996-2002, postoperative complications were observed in 2 cases (5.9%) which shows a similarity between their reported results and ours (14). 

According to the study conducted by Dr. Karimi and his colleagues in Tehran University in 2005, Stapedectomy was introduced as a more desired method in comparison to Stapedotomy in improving ABG incision (17).

## Conclusion

Regarding the obtained results, the frequency of otosclerosis in Iran is as high as other countries worldwide. Women are affected more than men and just like other similar studies, the most common involved age was 20-40 years. Most cases were affected with unilateral otosclerosis which is in contrast to other studies. More accurate investigations on a larger group of patients with different ethnicities are required in this regard. The number of studied parameters should also be increased. More extensive and accurate patients' data collection is also highly recommendable. Moreover, it should be kept in mind that the noticeable improvement in a patient's hearing is the direct result of a successful surgical operation.
